# Mental health and visual acuity in patients with age-related macular degeneration

**DOI:** 10.1186/s12886-022-02602-9

**Published:** 2022-10-02

**Authors:** Cheryl N. Fonteh, Marc T. Mathias, Naresh Mandava, Niranjan Manoharan, Anne M. Lynch, Roxanne Navo, Jennifer L. Patnaik, The University of Colorado Retina Research Group, Melanie Akau, Karen L. Christopher, Richard Davidson, Ruth T. Eshete, C. Rob Graef, Scott Hauswirth, Anne M. Lynch, Scott N. Oliver, Jeffery L. Olson, Alan G. Palestine, Jesse M. Smith, Brandie D. Wagner

**Affiliations:** 1 Department of Ophthalmology, University of Colorado School of Medicine, Aurora, CO USA; 2 Division of Ophthalmic Epidemiology, Department of Ophthalmology, University of Colorado School of Medicine, Mail Stop F731, 1675 Aurora Court, Aurora, CO 80045 USA

**Keywords:** Age-related macular degeneration, Mental health, Retina, Vison function questionnaires

## Abstract

**Background:**

Visual acuity (VA) loss has been associated with depression in patients with age-related macular degeneration (AMD). However, previous studies did not incorporate subgroups of AMD when correlating VA and mental health. The goal of this study was to describe the relationship between VA and mental health questions in patients with different classifications of AMD, and to identify associations of mental health subscale scores.

**Methods:**

AMD patients classified by multi-modal imaging were recruited into an AMD registry. Habitual VA was obtained by ophthalmic technicians using the Snellen VA at distance. At enrollment, patients completed the NEI-VFQ-25, which includes 25 questions regarding the patient’s visual functionality. Median with interquartile-range (IQR) scores on the mental health subscale of the VFQ were calculated by AMD classification and VA groups. Univariate and multivariable general linear models were used to estimate associations between mental health scores and variables of interest.

**Results:**

Eight hundred seventy-five patients were included in the study. Patients with bilateral geographic atrophy (GA) or bilateral GA and neovascular (NV) AMD scored lowest on the mental health subscales with a median (IQR) of 58.2 (38–88) and 59.3 (38–88). When stratified by VA, patients with a habitual VA of 20/200 or worse scored the lowest on mental health subscales scores: median of 43.8 (IQR: 31–62). Patients with a VA of 20/20 scored the highest: 87.5 (IQR: 81–94). Habitual VA of the better- and worse-seeing eye and AMD classification were significantly associated with mental health subscale scores (all *p* < 0.0001 in both the univariate and multivariable analysis, except the VA of the worse-seeing eye in multivariable model *p* = 0.027). Patients enrolled during the COVID pandemic had mental health scores that were 2.7 points lower than prior to the pandemic, but this difference was not significant in univariate (*p* = 0.300) or multivariable analysis (*p* = 0.202).

**Conclusion:**

There is a significant association between mental health questionnaire scores and AMD classification, as well as VA in both the better and worse-seeing eyes in patients with AMD. It is important for clinicians to recognize feelings of worry/ frustration in these patients, so they can be appropriately referred, screened, and treated for mental health problems.

## Background

Age-related macular degeneration (AMD) a disease of the photoreceptor support system is a chronic progressive disease affecting central vision, due to death of the photoreceptors caused by loss of the retinal pigment epithelium [1, 2]. AMD is one of the leading causes of low vision/ legal blindness in older populations [1]. Worldwide, over 196 million people are currently affected with AMD and the number is projected to increase to 288 million in 2040 [1]. As defined by Beckman Initiative for Macular Research Classification Committee AMD can be clinically divided into three stages: early, intermediate, and advanced [3]. The advanced stages can be further divided into neovascular AMD, geographic atrophy, or both [3, 4].

Symptoms of AMD can include difficulty recognizing faces, reading text, and performing housework [5]. These may directly lead to increased disability, placing AMD patients at an increased risk for mental health issues like clinical depression and anxiety [5, 6]. Furthermore, a person who is aware of gradual loss of vision may develop feelings of anxiety, loss of independence, insecurity and changes in social functioning that can lead to depression [7]. The negative impact of vision loss on psychological functioning, quality of life and social interaction has been reported in several studies [7–9]. Visually impaired individuals have a higher prevalence of depression compared to the general population [7]. Amongst older adults with visual impairment, patients with AMD have been shown to be at an increased risk for depression in comparison to patients with other eye diseases [6].

Cimarolli et al. summarized that patients with AMD had increased risk for developing depression and anxiety [6]. A few studies have shown an association between increasing AMD severity and higher prevalence of depression as reviewed by Dawson et al. [5] However, many of these studies did not differentiate between the types of AMD [5]. Using the Visual Function Questionnaire (VFQ)-25, a questionnaire sponsored by the National Eye Institute (NEI), [10] Patnaik et al. reported the highest mean composite scores for AMD patients in the early/ intermediate group, relatively high scores among patients diagnosed with neovascular (NV) AMD but not geographic atrophy (GA) AMD, and lower scores among GA AMD patients [11]. Concerning mental health questions, patients with bilateral advanced disease had lower scores in comparison to patients with unilateral disease [11].

As reviewed by Taylor et. al, increasing visual acuity (VA) loss is strongly correlated with depression in AMD [12]. Some studies have shown an association between depressive symptoms and loss of visual function regardless of VA level [13–15]. Prior publications examining mental health and VA in AMD patients have not distinguished between the phenotypes of AMD (early/intermediate, and unilateral and bilateral NV and GA). Patnaik et al., distinguished between the types of AMD when looking at mental health, but did not focus on associations of mental health or include VA in the analysis [11]. Using the same database and questionnaire as Patnaik et al., [11] the purpose of this study was to describe the relationship between VA and mental health questions in patients with different classifications of AMD, and to identify associations of mental health subscale scores. In addition, we examined mental health subscale scores before and during COVID to assess the potential impact of the pandemic.

## Methods

All eligible patients attending the Sue-Anschutz Eye Center were invited to participate and patients providing consent were enrolled in the study. Patients seen at our institution with a diagnosis of AMD were recruited and provided written consent for inclusion into an AMD registry developed by the Department of Ophthalmology at the University of Colorado School of Medicine. The goal of the registry was to develop a state-of-the art clinical database linked with biological samples and image data of patients with AMD. Patients with AMD who were recruited between July 9, 2014, and December 6, 2021, were included in the analysis. The goal of the registry was to develop a state-of-the art clinical database linked with biological samples and image data of patients with AMD. Patients who had ocular comorbidities, were terminally ill, have any active inflammatory disease or any other severe decrease in VA secondary to preexisting severe retina disease were excluded from the registry. Demographic and clinical characteristics were obtained via medical chart review and interview at the time of enrollment. [11, 16] Details of the methods of this registry are described elsewhere by Lynch et al. [17, 18] This study was approved by the Colorado Multiple Institutional Review Board.

### Classification of age-relate macular degeneration

Two vitreo-retinal specialists reviewed, graded, and documented multimodal retinal images according to the classification by Beckman Initiative for Macular Research Classification Committee [3]. A third specialist resolved discrepancies. After the AMD images were formally read and classified according to the Beckman Initiative, we further divided grouped the patients into the following groups for analysis: unilateral and bilateral early/intermediate, unilateral neovascular, bilateral neovascular, unilateral geographic atrophy, bilateral geographic atrophy, unilateral both advanced forms, and bilateral both advanced forms. This was done because patients often have different severity of disease in each eye and sometimes GA and NV in both eyes. NV AMD was defined as the presence of choroidal neovascularization in one or both eyes with no evidence of GA in either eye based on multimodal imaging (optical coherence tomography, color fundus photography, and autofluorescence). GA was defined as hypoautofluorescent atrophy of at least a quarter of the disc area on imaging, which reflected cell loss in the retinal pigment epithelium and outer retina [3]. Furthermore, GA was diagnosed based on the presence of complete retinal epithelium and outer retinal atrophy (cRORA) using multimodal imaging. The following specific OCT criteria used to diagnose cRORA; (i) an area of hyper transmission of at least 250 µm, (ii) a zone of disruption or attenuation of the retinal pigment epithelium (RPE) of at least 250 µm in diameter, (iii) absence of any signs of RPE tear and (iv) evidence of overlying degeneration of the photoreceptor [19]. Intermediate AMD was defined as large drusen > 125 µm and/or any AMD pigmentary abnormalities. Early AMD was defined as medium drusen > 63 µm and less than or equal to 125 µm and no AMD pigmentary abnormalities [3].

### Habitual Visual Acuity (HVA)

At the time of enrollment into the study, habitual VA was obtained in the clinic by a trained certified ophthalmic technician using the Snellen VA charts for distance viewing, as is standard of care. For statistical analysis, Snellen equivalent measures are provided for the median logarithm of the minimum angle of resolution (logMAR) values for each group. The better-seeing eye was defined as the eye with the lower logMAR measure for each patient. The worse-seeing eye demonstrated equal, or higher logMAR VA. Snellen equivalent measures are provided for logMAR values [16]. VA groups were categorized as 20/20 or better, 20/25 and 20/30, 20/40, 20/50 and 20/60, 20/70 to 20/160, and 20/200 or worse.

### Questionnaire

A paper copy of the 25-item NEI-VFQ-25 was either self-administered by patients enrolled in the study or administered by professional research assistants. The 25-item NEI-VFQ-25 is a visual questionnaire that includes 25 questions regarding the patient’s visual functionality with regular daily activities [10]. The NEI-VFQ was developed to measure a patient’s subjective assessment of visual function. It has been validated in patients with low vision caused by multiple diseases, including primary open-angle glaucoma, cataracts, diabetic retinopathy, and AMD [10]. Four questions in the subscale regarding mental health were the focus of the current analysis. These questions include, “*How much of the time do you worry about your eyesight?”, “I feel frustrated a lot of the time because of my eyesight”, “I have much less-control over what I do, because of my eyesight”, “I worry about doing things that will embarrass myself or others, because of my eyesight.”* For each of the four questions, there were answer options that differed and were all on 5-point Likert scale which were scored with values of 0, 25, 50, 75, and 100 with high scores representing the better visual functionality score.

### Statistical analysis

Descriptive statistics included percentages for categorical variables and means, medians, standard deviations (SD) and inter-quartile ranges (IQR) for continuous variables. Questions regarding mental health were combined to obtain a mental health subscale score and compared across AMD classification groups and HVA groups for the better- and worse-seeing eyes with box plots. In addition, gender, age, marital status, and a binary variable of before the COVID pandemic (defined as patients enrolled before March 15, 2020) and during the COVID pandemic (defined as patients enrolled after March 15, 2020) were assessed to determine associations with patient reported mental health subscale scores. Patient-level univariate and multivariable general linear models were utilized to estimate associations between mental health subscale scores and variables of interest. P-values less than 0.05 were noted as statistically significant. Analysis was performed using SAS software version 9.4 (SAS Institute, Inc, Cary, NC).

## Results

**Fig. 1:**
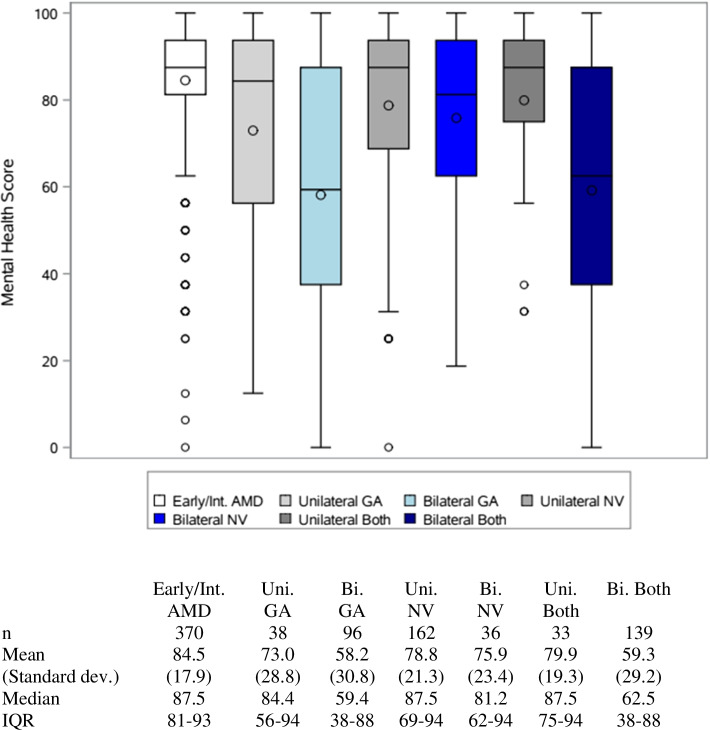
Box plots of mental health subscale scores by AMD classification. The line in each boxplot represents the median and the circle correpsond to the mean values. The boxplots extend to the 25^th^ and 75^th^ percentiles, and the whiskers extend to a maximum of 1.5 IQR. IQR = inter-quartile range

The initial cohort was comprised of 967 patients and 44 were excluded due to uncertain or unreadable AMD classification following image review and an additional 48 patients were excluded due to not completing the VFQ-25. A total of 875 patients were included in the study. Table 1 illustrates patient demographics and select clinical characteristics. The majority of patients were white (94.7%) and 60.1% were female. Table 2 shows a detailed description of AMD classification groups, number of patients in each group, and median HVA. Among patients with any form of NV AMD, 87.8% were being treated with anti-VEGF injections. Boxplots of mental health subscale scores by AMD classification are demonstrated in Fig. 1. Patients with early/intermediate AMD had the highest scores with a median and IQR of 87.5 (81–93). Patients with bilateral GA and bilateral both advanced had the lowest scores of 59.4 (IQR: 38–88) and 62.5 (IQR: 38–88), respectively.

**Table 1:** Demographic and clinical characteristics of the study cohort by AMD classification

	**Early/Int. AMD**	**Advanced AMD**		
	**eiAMD**	**GA**	**NV AMD**	**Both Advanced**^a^
**Number of Patients**	370	135	198	172
**Female gender**	61.4%	57.0%	58.6%	61.6%
Age, Mean (SD)^**^	75.2 (11.4)	81.1 (9.8)	78.0 (10.1)	81.7 (7.9)
**Race/ethnicity**				
**White**	352 (95.1%)	128 (94.8%)	186 (93.9%)	163 (94.8%)
**Hispanic**	6 (1.6%)	5 (3.7%)	5 (2.5%)	3 (1.7%)
**African American**	4 (1.1%)	1 (0.7%)	3 (1.5%)	3 (1.7%)
**Asian**	3 (0.8%)	1 (0.7%)	1 (0.5%)	3 (1.7%)
**Other**	5 (1.4%)	0 (0%)	3 (1.5%)	0 (0%)
**Married**^ǂ^	127 (34.6%)	49 (36.3%)	74 (38.1%)	78 (45.9%)
Bilateral advanced stage^**^	-	71.8%	18.2%	80.8%
Lens status^**^				
**Pseudophakic, both**	205 (55.4%)	96 (71.1%)	109 (55.0%)	114 (66.3%)
**Pseudophakic, one**	19 (5.1%)	9 (6.7%)	18 (9.1%)	19 (11.0%)
**Cataract, both**	142 (38.4%)	30 (22.2%)	70 (35.4%)	38 (22.1%)
**Phakic, both**	4 (1.1%)	0 (0%)	1 (0.5%)	1 (0.6%)
HVA in better-seeing eye^**^				
**Mean (SD) LogMAR**	0.096 (0.12)	0.379 (0.31)	0.165 (0.20)	0.430 (0.46)
**Median LogMAR**	0.097	0.301	0.097	0.301
**Median Snellen**	20/25	20/40	20/25	20/40
HVA in worse-seeing eye^**^				
**Mean (SD) LogMAR**	0.265 (0.34)	0.800 (0.73)	0.699 (0.81)	1.253 (1.01)
**Median LogMAR**	0.176	0.602	0.398	0.835
**Median Snellen**	20/30	20/80	20/50	20/140

HVA is habitual visual acuity

^a^ The number of patients with the various combinations included in the Both Advanced groups are described in detail in Table 2

^**^ Significantly different across groups, *p* < 0.05

^ǂ ^*p* < 0.10 across groups

**Table 2:** Median HVA and number of eyes for subgroups based on AMD classification

	Median HVA		Detailed Description	# Patients
AMD classification and eyes affected	Better-seeing eye	Worse-seeing eye		
Early/Intermediate	20/25	20/30	Total	370
			One eye early	11
			Both eyes early	44
			One eye early and one eye intermediate	35
			One eye intermediate	13
			Both eyes intermediate	267
Unilateral NV	20/25	20/50	NV in one eye and GA in neither	162
Bilateral NV	20/25	20/60	NV in both eyes and GA in neither	36
Unilateral GA	20/30	20/40	GA in one eye and NV in neither	38
Bilateral GA	20/50	20/100	GA in both eyes and NV in neither	97
Unilateral both advanced	20/25	20/80	One affected eye has both NV and GA	33
Bilateral both advanced	20/50	20/150	Total	139
			One eye NV and one eye GA	11
			Both eyes GA and one eye NV	37
			Both eyes NV and one eye GA	24
			Both eyes GA and NV	67

*HVA* Habitual visual acuity, *AMD* Age-related macular degeneration, *NV* Neovascular, *GA* Geographic atrophy

**Fig. 2:**
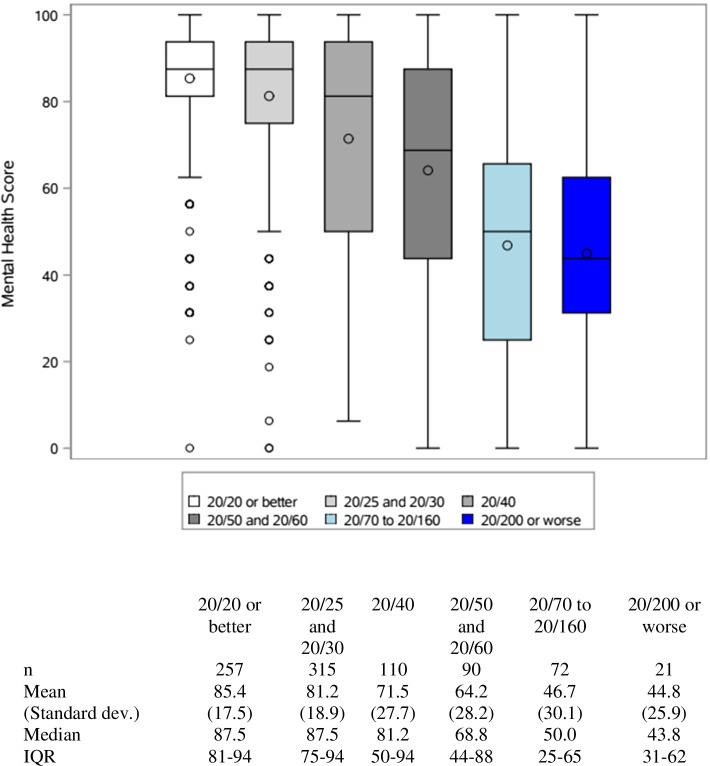
Box plots of mental health subscale scores by HVA in better-seing eye. The line in each boxplot represents the median and the circle correpsond to the mean values. The boxplots extend to the 25^th^ and 75^th^ percentiles, and the whiskers extend to a maximum of 1.5 IQR. IQR = inter-quartile range

**Fig. 3:**
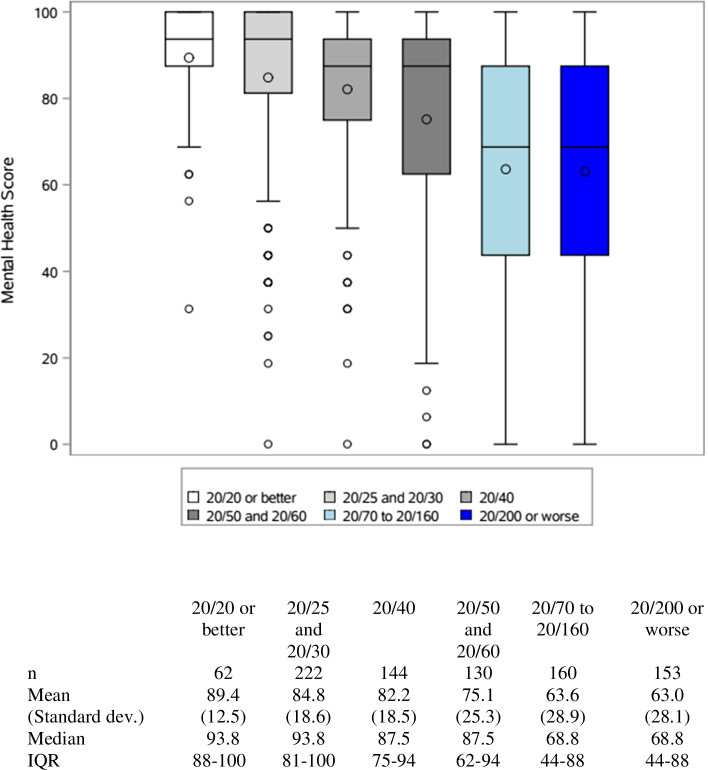
Box plots of mental health subscale scores by BCVA in worse-seing eye. The line in each boxplot represents the median and the circle correpsond to the mean values. The boxplots extend to the 25^th^ and 75^th^ percentiles, and the whiskers extend to a maximum of 1.5 IQR. IQR = inter-quartile range

Boxplots of mental health subscale scores by HVA in the better-seeing eye are portrayed in Fig. 2. Patients with an HVA of 20/20 or better had the highest median score of 87.5 (IQR: 81–94). Patients with an HVA of 20/200 or worse had the lowest median score of 43.8 (IQR: 31–62). On the other hand, Fig. 3 illustrates boxplot of mental subscale scores by HVA in the worse-seeing eye. Similarly, patients with an HVA of 20/20 or better had the highest median score of 93.8 (IQR: 88–100). While patients with an HVA of 20/70 or worse had the lowest median score of 68.8. There is a stepwise decline in mental subscale scores as HVA worsens in the better seeing eye, and there is also an incremental decrease in mental subscale scores as HVA worsens in the worse seeing eye.

**Fig. 4:**
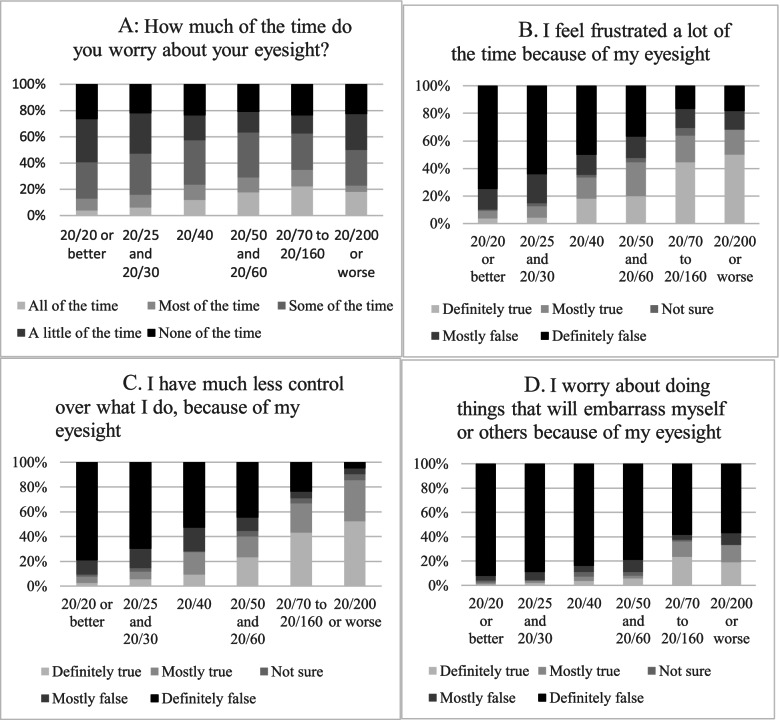
Individual mental health subscale question responses by visual acuity in the better-seeing eye

Figure 4 displays percentage of responses to each question on the mental health subscale score across different HVAs. As shown in Fig. 4A, the percentage of patients who worry about their eyesight all of the time increases with worse HVA in the better-seeing eye, yet the percentage of people who worry none of the time remains fairly stable across HVA categories. Figure 4B, C and D illustrate that as HVA worsens, patients increasingly “I feel frustrated a lot of the time because of eyesight”, “I have much less control over what I do, because of eyesight”, and “I worry about doing things that will embarrass myself or others because of my eyesight”. “Feeling frustrated” and “Having much less control over what I do because of my eyesight” had the highest increase as HVA worsened (Figs. 4B and C).

Univariate and multivariable analysis of associations of mental health subscale score among AMD patients are shown in Table 3. In the univariate analysis, logMAR of the better seeing eye, logMAR of the worse seeing eye, and AMD classification were significant associations of mental health subscale scores, all with *p*-values < 0.0001. In addition, being unmarried was significantly associated with lower mental health subscale score (*p* = 0.0002). Female sex, age and COVID time period were not significantly associated with mental health subscale scores in univariate analysis. In the multivariable analysis: worse HVA of the better seeing eye was significantly associated with worse mental health scores (PE = -23.0 (SE 3.2), *p* < 0.0001). Worse HVA of the worse seeing eye was significantly associated with worse mental health scores (PE -2.7 (SE 1.2), *p* = 0.027). Worse mental health scores were also significantly associated with increasing age (PE -0.3 (SE 0.08), *p* < 0.0001), and more advanced forms of AMD (*p* < 0.0001). Married participants had significantly higher mental health scores in comparison to those not married (PE 4.0 (SE 1.7), *p* = 0.017). Patients enrolled during the COVID pandemic had mental health scores that were 2.7 points lower than prior to the pandemic, although this difference was not significant in the univariate (*p* = 0.300) or multivariable analysis (*p* = 0.202). Thus, more advanced AMD classification, worse HVA in the better seeing eye, worse HVA in the worse seeing eye, older age and not being married were associated with worse mental health scores in the final model.

**Table 3:** Univariate and multivariable analysis of associated effect of mental health scores among AMD patients

	Univariate Analysis		Multivariable Analysis	
	PE (SE)	*p*-value	PE (SE)	*p*-value
AMD Classification				
Early/Int	Reference		Reference	
Unilateral NV	-5.7 (2.2)	< 0.0001	-4.0 (2.1)	< 0.0001
Bilateral NV	-8.6 (4.0)		-4.3 (4.0)	
Unilateral GA	-11.5 (3.9)		-9.0 (3.8)	
Bilateral GA	-26.3 (2.6)		-19.3 (2.7)	
Unilateral Both	-4.6 (4.2)		-2.7 (4.0)	
Bilateral Both	-25.2 (2.3)		-14.9 (2.7)	
Female Sex	-3.0 (1.7)	0.084	-1.4 (1.6)	0.389
Age (years)	-0.1 (0.08)	0.250	-0.3 (0.08)	< 0.0001
Married	6.7 (1.8)	0.0002	4.0 (1.7)	0.017
HVA for Better-seeing Eye (logMAR)	-36.9 (2.6)	< 0.0001	-23.0 (3.2)	< 0.0001
HVA for Worse-seeing Eye (logMAR)	-10.1 (1.0)	< 0.0001	-2.7 (1.2)	0.027
During COVID pandemic	-2.7 (2.6)	0.300	-3.1 (2.4)	0.202

*PE* Parameter estimate, *SE* Standard error, *HVA* Habitual visual acuity, *AMD* Age-related macular degeneration, *NV* Neovascular, *GA* Geographic atrophy

## Discussion

In this study, we demonstrate a significant association between mental health questionnaire scores and AMD classification, as well as VA in both the better and worse-seeing eyes in patients with AMD. Patients with bilateral GA and bilateral both advanced AMD had the lowest mental health subscale scores. Patients with HVA 20/20 or better in both the better and worse seeing eye had the highest mental scores, while patients with 20/200 or worse in both the better and worse seeing eye had the lowest mental health score. In addition to the HVA in the better seeing eye and worse seeing eye, AMD classification and age were also significantly associated with mental health subscale scores in our cohort. Although enrollment of patients during the COVID pandemic was not a significant association, patients enrolled during the pandemic had a mean score that was 2.7 points lower than prior to the pandemic.

The NEI VFQ-25 was developed to provide an estimation of vision-targeted-health-related quality of life from the patient’s perspective for use to evaluate the outcomes of eye disease intervention [10]. Although the NEI VFQ-25 cannot be used to diagnose mental health problems, such as depression and anxiety, a significant association has been found between depression and reduced NEI VFQ-25 scores [20]. A study from Submacular Surgery Trial Research Group suggests that a 5-point change in the individual subscale score in the NEI-VFQ and a 4-point change in the overall score may be considered the minimum for clinically significant changes over time [21]. Thus, lower VFQ-25 scores, particularly the mental health subscales questions, may be an indicator for depression. Our results show patients with bilateral GA and bilateral both advanced types of AMD scored lowest on the mental health subscale scores. These findings were in keeping with the findings of Patnaik et al., given data was obtained from the same AMD registry [11]. Likewise, patients diagnosed with only NV AMD had relatively higher scores in comparison to GA patients [11]. Taking these results, a step further, we identified associations of the VFQ-25 mental health subscale score among AMD patients.

Depression is one of the most common chronic medical conditions in older adulthood [22]. Major depression has a prevalence of 1–4% of the general elderly population, with an equivalent incidence of 0.15% per year. Both its prevalence and incidence double after age 70–85 years [23]. There are several factors that contribute to the higher prevalence of depression in the elderly. Some include psychosocial adversity, increased prevalence of chronic medical illnesses and cognitive impairment, ageing-related and disease-related processes that can compromise the integrity of the hippocampus, amygdala, frontostriatal pathways increasing vulnerability to depression [23]. Thus, as patients get older, they are more susceptible to these factors and more likely to develop symptoms of depression, which can explain why age is inversely associated with the mental health score. In this study, marital status had a significant association with VFQ-25 scores in both the univariate and multivariable analysis. These results are in agreement with a meta-analysis study by Yan et al. which showed that unmarried status is a risk factor for depression in older adults [24].

The mental health VFQ-25 scores in our study declined by AMD classification after adjusting for VA. Medical therapy can be used to maintain vision among NV AMD (intra-vitreal anti-VEGF injections), while there is no established treatment for GA [6]. Moreover, in patients with early/ intermediate AMD, several therapeutic approaches (smoking cessation, a healthy diet, antioxidants) can be taken to slow progression of the disease to more advanced forms [4]. Thus, the benefits of these therapies in patients with early forms of AMD/NV AMD versus lack of any therapy in GA patients are likely contributing to AMD classification as a significant association of mental health VFQ-25 scores [11].

Vision is important for enjoyment of life and activities of daily living; thus, it is logical that vision loss would have a profound effect on mental health [7], making VA a significant association of mental health score. However, there is conflicting literature examining the relationship between severity of vision loss and depression as reviewed by Casten et. al [12, 25]. Proposed mechanisms linking depression and AMD include loss of the ability to pursue valued activities, physical limitation, and social isolation [25]. Some studies suggest that patients with AMD are at higher risk of developing depression, regardless of VA, because knowledge of having a non-treatable, progressive disabling disease like AMD can act as a stressor and induce depression [15, 25]. Our study is novel because it shows a stepwise decline of the mental health subscale score with worsening vision in both the better seeing and worse seeing eye. As expected, patients with HVA 20/200 or worse had the lowest mental health scores when both the better- and worse-seeing eyes were analyzed separately. In comparing the mental health scores for the better and worse seeing eye at each BCVA category, the better-seeing eye had higher parameter estimates in both the univariate and multivariable analysis indicating a stronger impact on mental health scores for the better-seeing eye. In a small pilot study, Podbielski et al. showed that vision in the worse seeing eye is not as bad as it seems when compared to the better-seeing eye (ie the worse seeing eye does not have as much impact on overall visual functioning) [26]. Residual or partial vision is described as when vision is not lost, but impaired, and easily subjectively reported by patients [27]. Because residual vision is not reliably measured by visual acuity, and in most cases the residual vision in the worse and better seeing eyes are not much different using modern outcome measures (visual acuity, fixation stability, preferred retina loci topography/span) [26]. This could explain why the mental health scores were higher in patients with worse-seeing eyes at each HVA category. Patients with AMD in our institution are not refracted at each visit, therefore, HVA at time of enrollment was used for this study. Since most of these patients are receiving regular care for their AMD diagnosis, we do not expect HVA and BCVA to substantially differ.

Government and individual responses to the COVID-19 pandemic affected the lives of millions of people worldwide and changed ways of living, studying, working, and socializing [28]. COVID-19 has resulted in higher prevalence of mental health problems, particularly in patients with noninfectious chronic diseases [28]. Elderly patients are more likely to have chronic diseases, putting them at increased risk of mental health problems during the pandemic. Several studies have demonstrated the protective effects of social participation in the health of the elderly, and it is suggested that social distancing for COVID-19 negatively affected the mental and physical health in older people [29]. In our cohort, there was a 2.7-point lower mean mental health subscale score seen in patients enrolled during the pandemic, however, this difference did not reach statistical significance. It is important to note that based on published sample size estimate calculations, 2858 study participants would be required to detect a two-point difference in the mental health subscale scores of the VFQ-25 [10]. The difference shown here, although not significant, reiterates findings from other studies which show COVID has a negative impact on mental health, particularly in the older patients/ patients with noninfectious chronic diseases [28, 29]. It is important for health care providers to be aware of this, so medical monitoring, and treatment for patients with chronic diseases can be preoptimized during the pandemic [28].

The first limitation in this study is the cross-sectional design does not assess patient-level changes associated with disease progression. Patients have been recruited in this study since 2014 when the study began and can be recruited at any point in their disease duration. The mental health scores and VA can change as the disease progresses. Future research at our institution will track VA and mental health scores as the disease progresses. Secondly, mental health scores can be influenced by much more than AMD diagnosis, including other ocular and system comorbidities, as well as physical functioning abilities. Thirdly, VFQ scores are self-reported by the patient and not sufficient to make a diagnosis of depression or other specific mental health conditions. There are also other mental health specific questionnaires which could more fully assess mental health status; however, the VFQ-25 is the typical survey administered in our AMD clinics. We will consider using other well-studied screening depression questionnaires (such as the CES-D and geriatric Depression Scale) for future studies. Fourthly, some of the AMD groups in this study have a small sample size which may not provide generalized results. There is evidence that early stages of AMD are associated poor rod-mediated vision and patients have difficulty performing tasks under low luminance [30]. Thus, another limitation is patients’ difficulty with task was analyzed under low luminance. The strengths in this study include a large cohort of AMD patients, very careful image review and classification of AMD into separate groupings and the ability to compare NEI VFQ-25 mental health questions results by categories of VA.

## Conclusion

In summary, we report the associations of mental health questions on the NEI VFQ-25 in patients with AMD, as well as show a correlation between VA and mental health scores in both the better and worse seeing eyes. Age, AMD classification, VA and marital status play a role in predicting mental health scores as measured by the VFQ-25 mental health subscale. It is important for clinicians to recognize feelings of worry/ frustration in at-risk patients, so they can be appropriately screened, referred, and treated for mental health problems.

## Data Availability

The datasets generated and/or analyzed during the current study are not publicly available due not having been approved by our IRB but are available from the corresponding author on reasonable request.

## Abbreviations

AMD: Age related macular degeneration
GA: Geographic atrophy
NV: Neovascular
VA: Visual acuity
HVA: Habitual visual acuity
VFQ: Visual Function Questionnaire
NEI: National Eye Institute
MAR: Minimum angle of resolution
SD: Standard deviations
IQR: Inter-quartile range
